# Dr. Todd

**Published:** 1840-10

**Authors:** 


LIBRARY OF THE

Orleans Parish Medical Society

DR. TODD.

DIED on the 4th August, at the village of Hurst, in Sussex, TWEEDIE JOHN TODD, M.D., for many years one of the principal physicians of Brighton. Dr. Todd was born at Berwick in the year 1789, his father being treasurer of the corporation of that town. He received his classical education in his native town, whence he proceeded, at an early age, to study medicine at Edinburgh. He did not, however, remain to graduate, having accepted the appointment of assistant-surgeon in the navy in the year 1809. He passed the first eighteen months of his service in the improving field of the great naval hospital at Plymouth, where he already gave promise of his future excellence as a practitioner. Shortly after going afloat he was promoted to the rank of surgeon by the present commander in chief in the Mediterranean, Admiral Sir Robert Stopford, who entertained so high an opinion of Dr. Todd that, although then only in his twenty-third year, he appointed him to his own flag-ship. In this capacity he served several years, chiefly in the East Indies and at the Cape of Good Hope, where he made his experiments on Torpedo Electricus, afterwards published in the Philosophical Transactions. On retiring from the navy in 1816, Dr. Todd went abroad, visiting various places on the continent, and, before his return, spending six or seven years consecutively in Italy. During part of this time, he travelled as physician with the late Marchioness of Bute, and remained with her for some time after her return to England. In the year 1829 he settled at Brighton, and speedily came into very extensive practice, which he retained until within six months of his death, when he was attacked by tuberculous pleurisy, which proved fatal after confining him for many months to his bed. Dr. Todd obtained his first degree as a physician at Montpelier during his residence abroad, and he took a second at Aberdeen previously to settling at Brighton. He was prevented taking his degree at Edinburgh, as he wished, from not having completed the full measure of study required by the statutes of the University; but before receiving the doctorate at Aberdeen he visited that city, and underwent an examination by the medical faculty of the University.

Dr. Todd was a man of very considerable learning and of great talents. His knowledge of his profession as a science was profound, and he had the happy and rare talent of making his scientific knowledge habitually subservient to his practice of medicine as an art. This, added to his possession of that tact "unteachable untaught," without which mere learning is of slight avail in practical medicine, rendered him one of the most skilful and successful among his contemporary physicians. In his views of practice he combined most happily the rational expectation or trust in nature of the Hippocratic school with the traditionary wisdom and fearless energy of British empiricism, blending the whole, by his intimate knowledge of physiology and pathology, into a scientific code of therapeutics which led to the most successful results. In accordance with this system he trusted fully as much to regimen as to medicaments; and of this last class of agents he greatly prized mineral waters, being conversant with their true mode of action and proper application to a degree we believe rare among the practitioners of this country.

Dr. Todd was the author of several papers on natural history, published in the Transactions of the Royal Society and the Journal of Science—On the Torpedo, on the Regeneration of parts in the Aquatic Salamander,—On the luminous power of some of the Lampyrides, &c. He was the author of the elaborate article *Indigestion* in the Cyclopædia of Medicine, a treatise strikingly exhibiting his knowledge of what had been done by others, his accuracy and minuteness of observation, as well as his scientific knowledge and practical skill. This treatise we regard as by far the best that has yet appeared on the subject. The only separate work published by Dr. Todd was that which he named *The Book of Analysis*, and which appeared in 1831. In this small volume, which he also entitled *A New Method of Experience*, he endeavoured to point out the means of applying the Baconian induction to Medicine and the other natural sciences, and he succeeded in this attempt to a degree which only those who have examined the work with attention can fully appreciate. He labours here in the same field as Louis, but altogether independently of that great physician, who, we are convinced, would be one of the foremost in admitting that Dr.

Todd was the contemporaneous discoverer and promulgator of *a numerical method*, if not exactly similar to, of the same nature and importance as his own. The means by which Dr. Todd proposed to improve both the science and practice of medicine are "comprehended," he says, "in a few words—true, distinct, circumstantial *observation*—clear, severe, searching *analysis*;" and he planned a series of tables and signs for carrying out his numerical method, which are no less ingenious than philosophical. The Book of Analysis has not yet gathered all its fame; and we ourselves hope, ere long, fully to discuss its merits in our pages.

Dr. Todd had been engaged for many years in a series of microscopical researches on living animals illustrative of different parts of physiological and pathological science, and especially of the processes employed by nature in the healing and regeneration of wounded and lost parts. His labour has not all perished with him, although we fear he has left on record few of the generalized results of his observations and experiments. He has, however, left a large collection of beautiful microscopical preparations, which we trust may yet be rendered fruitful to science in the hands of some kindred spirit, and thus prove the best and most characteristic memorial and monument of their lamented author.

Dr. Todd was a man of high moral excellence, of the strictest honour and integrity, of an amiable temper, and most pleasing manners. He was no less beloved than respected by his numerous friends and patients.

